# A deep-learning-based model for assessment of autoimmune hepatitis from histology: AI(H)

**DOI:** 10.1007/s00428-024-03841-5

**Published:** 2024-06-15

**Authors:** Caner Ercan, Kattayoun Kordy, Anna Knuuttila, Xiaofei Zhou, Darshan Kumar, Ville Koponen, Peter Mesenbrink, Serenella Eppenberger-Castori, Parisa Amini, Marcos C. Pedrosa, Luigi M. Terracciano

**Affiliations:** 1 Institute of Pathology and Medical Genetics, University Hospital Basel, University of Basel, Schönbeinstrasse 40 4056, Basel, Switzerland; 2 Novartis Pharmaceuticals Corporation, East Hanover, NJ USA; 3 Aiforia Technologies PLC, Helsinki, Finland; 4 Novartis Institutes for BioMedical Research, Basel, Switzerland; 5 Novartis Pharma AG, Basel, Switzerland; 6 Department of Biomedical Sciences, Humanitas University, Pieve Emanuele, Milan, Italy; 7 IRCCS Humanitas Research Hospital, Rozzano, Milan, Italy

**Keywords:** Artificial intelligence, Deep-learning, Autoimmune hepatitis, AIH, Computational pathology

## Abstract

Histological assessment of autoimmune hepatitis (AIH) is challenging. As one of the possible results of these challenges, nonclassical features such as bile-duct injury stays understudied in AIH. We aim to develop a deep learning tool (artificial intelligence for autoimmune hepatitis [AI(H)]) that analyzes the liver biopsies and provides reproducible, quantifiable, and interpretable results directly from routine pathology slides. A total of 123 pre-treatment liver biopsies, whole-slide images with confirmed AIH diagnosis from the archives of the Institute of Pathology at University Hospital Basel, were used to train several convolutional neural network models in the Aiforia artificial intelligence (AI) platform. The performance of AI models was evaluated on independent test set slides against pathologist’s manual annotations. The AI models were 99.4%, 88.0%, 83.9%, 81.7%, and 79.2% accurate (ratios of correct predictions) for tissue detection, liver microanatomy, necroinflammation features, bile duct damage detection, and portal inflammation detection, respectively, on hematoxylin and eosin-stained slides. Additionally, the immune cells model could detect and classify different immune cells (lymphocyte, plasma cell, macrophage, eosinophil, and neutrophil) with 72.4% accuracy. On Sirius red-stained slides, the test accuracies were 99.4%, 94.0%, and 87.6% for tissue detection, liver microanatomy, and fibrosis detection, respectively. Additionally, AI(H) showed bile duct injury in 81 AIH cases (68.6%). The AI models were found to be accurate and efficient in predicting various morphological components of AIH biopsies. The computational analysis of biopsy slides provides detailed spatial and density data of immune cells in AIH landscape, which is difficult by manual counting. AI(H) can aid in improving the reproducibility of AIH biopsy assessment and bring new descriptive and quantitative aspects to AIH histology.

**Supplementary Information:**

The online version contains supplementary material available at 10.1007/s00428-024-03841-5.

## Introduction

Autoimmune hepatitis (AIH) is a chronic, immune-mediated, and progressive inflammatory rare liver disease, and diagnosis frequency is increasing worldwide [1]. Liver histology plays a critical role in diagnosis and is considered mandatory in the diagnostic protocols [2–4]. However, the numerous challenges associated with liver biopsy interpretation, including low inter-observer agreement, disease heterogeneity, and lack of specific histological features, impact accurate and consistent characterization of histology [5]. No specific histological feature exists for diagnosing AIH [6]. The histological features of AIH primarily include elementary lesions typically observed in various forms of chronic hepatitis [7]. Moreover, some studies have demonstrated in liver biopsies of AIH the presence of non-classical microscopic features, such as bile duct injury [8]. However, as these features have not been extensively studied, their prevalence and significance remain unclear. Thus, an effective AIH-focused image analysis tool to detect these relevant elementary lesions in chronic hepatitis biopsies is needed.

Artificial intelligence (AI) tools using convolutional neural networks (CNN) have demonstrated potential applications in medical imaging and pathological diagnostics [9]. Despite CNN’s promising performance in classification, detection, and quantification, usage of deep learning in clinical practice has not yet been adapted owing to its lack of interpretability [10–12]. Pathologists prefer clear declarative representations from AI models that they can perceive and comprehend in order to determine their decision boundaries [10]. It has been shown that the diagnostic performance of a pathologist is improved when they have visual output overlay of AI model’s predictions along with slide images [13]. Machine learning algorithms have been used in clinical prediction models with higher diagnostic accuracy for several liver diseases [14]. Furthermore, integrating and combining AI with digital pathology may reduce inter-observer variability [15, 16]. The multilayered complex landscape of AIH histology requires a hand-crafted pipeline. Instead of having a characteristic specific lesion (e.g., a ground-glass cell for HBV hepatitis [17] or a ballooning cell for steatohepatitis) [18], AIH biopsies have histological features of chronic hepatitis. This requires detection of various inflammation features, which are distinguished from each other by their locations and extensions. Therefore, we built a fully supervised multilayered pipeline by combining different computer vision models to achieve detection of various chronic hepatitis features.

This study aims to develop the first ever deep-learning AI tool (artificial intelligence for hepatitis, AI[H]) that evaluates and classifies different regions of AIH histology to provide a granular, quantifiable, and reproducible analysis of AIH pathology.

## Patients and methods

### Patient cohort and liver biopsies

A total of 123 pre-treatment liver biopsies from 116 anonymized adult patients with AIH, collected between 1996 and 2020, were selected from the archives of Institute of Pathology at University Hospital Basel, Basel, Switzerland. The diagnosis of AIH was confirmed according to current European Association for the Study of the Liver guidelines [19]. Patients who received a diagnosis of any other liver disease along with AIH at the time of diagnosis or during follow-up were excluded from the study. For the exploration of performance on other hepatitis samples, two liver biopsies with acute hepatitic histologic pattern drug-induced liver injury, four HBV hepatitis biopsies, and five HCV biopsies were scanned and analyzed by the trained model. Visual inspections were conducted to assess the results.

Written informed consent was obtained from all patients included in the study. The study protocol conformed to the ethical guidelines of the Swiss Federal Human Research Act (key ethical guidelines of January 2014) and was approved by the Ethics Committee of Northwestern Switzerland (authorization number: EKNZ 2014–362).

### Liver biopsies

#### Microscopical evaluation of samples

Hematoxylin and eosin (H&E) and Sirius red-stained slides from the University Hospital Basel Institute of Medical Genetics and Pathology’s biobank were used. Two experienced pathologists concurrently assessed the liver biopsies using a multiheaded microscope and employed the Ishak scoring system, a well-established framework for grading necro-inflammatory activity and fibrosis in chronic hepatitis histology [20], and consensus recommendations for histological criteria of AIH from the International AIH Pathology Group [6]. This collaborative approach ensured accurate and consistent evaluations, with the final scores representing the agreed-upon assessments by both pathologists.

#### Imaging and software

The digital whole-slide images (WSIs) of H&E-stained liver tissue needle biopsies were scanned at a magnification equivalent to 40 × objective with a Pannoramic SCAN II scanner and software (3DHISTECH™, Hungary) which produces a final digital slide with 0.24 µm/px resolution, and these slides were used for AI model development. The WSIs were uploaded to the Aiforia platform (Aiforia Technologies Plc, Helsinki, Finland), featuring deep learning and cloud-based image analysis technology.

#### Convolutional neural networks training: creating AI models via supervised learning

Models were trained using the Aiforia research cloud platform on WSIs. To develop the AI models, biopsies were randomly split into training (80%) and test (20%) datasets. Separate CNN-based AI models were used to predict different components of histological images, each focusing on a different feature of AIH pathology. H&E slides were used to train the CNNs for semantic segmentation and object-based detection of main liver microanatomy, hepatitis morphology, immune cells, and bile duct damage. The fibrosis grading model was trained using Sirius red slides. Training and test dataset annotations for the immune cell classification models were performed by two pathologists on digital slides; the rest of the annotations were performed by a single pathologist.

**Fig. 1:**
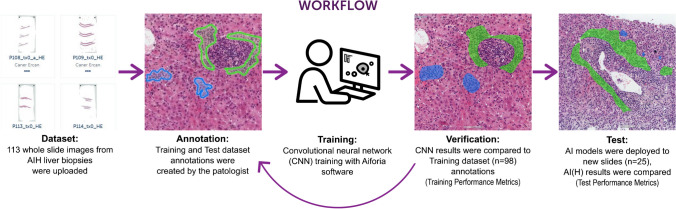
Workflow of AI(H) development. The dataset was divided into training and test subsets. Pathologist annotations were employed for training the model. Model performance was evaluated using performance metrics and visual overlays of predictions. Subsequently, the model’s performance was tested on a held-out subset of test slides to assess its generalizability. Blue, focal necrosis; Green, interface hepatitis; AI, artificial intelligence; AIH, autoimmune hepatitis; AI(H), artificial intelligence for hepatitis; CNN, convolutional neural network; n, number of patients

The liver microanatomy detection model was trained to segment liver tissue into portal area, lobular area, and central vein compartments. The necro-inflammation model was trained to identify focal necrosis, interface hepatitis, and confluent necrosis. The immune cell classification model was designed to detect, classify, and quantify five types of immune cells including lymphocytes, plasma cells, macrophages, eosinophils, and neutrophils, along with acidophil bodies. In the slides of the training dataset, representative morphological areas were selected for annotation (Fig. 1). Of the total training data set of images, a variable area per class was used for training. Table 1 provides the details for annotating the different image features in the training material (99 WSIs).

**Table 1:** Ground truth for AI model training

Layer	Staining	Class	Ground truth (included)	Background (excluded)
Tissue	H&E	Liver tissue	Liver tissue	Artifacts, white space outside tissue
Microanatomy	H&E	Portal area Lobular area Central vein	Normal liver microanatomic structures	Artifacts, white space outside tissue
Necroinflammation	H&E	Interface hepatitis Focal necrosis Focal confluent necrosis Perivenular necrosis Bridging necrosis Panacinar necrosis	The regions with chronic hepatitis related injury	Portal area and non-inflamed liver lobule
Portal inflammation	H&E	Mild Moderate Severe	Inflamed portal areas	Non-inflamed portal area and liver lobule
Inflammatory cells	H&E	Lymphocytes Plasma cells Macrophages Neutrophils Eosinophils Acidophil bodies	Immune cells	Other cell types, e.g., hepatocytes, fibroblasts, bile duct epithelium
Bile ducts	H&E	Damaged Normal	Damaged: Ducts with inflammatory infiltrate, dysplasia, disordered epithelium Normal: Uninjured bile duct epithelium	Ductular proliferation, large ducts, other cell types and artifacts
Tissue	Sirius red	Liver tissue	Liver tissue	Artifacts, white space outside tissue
Microanatomy	Sirius red	Portal area Central vein	Normal liver microanatomic structures	Artifacts, white space outside tissue
Fibrosis	Sirius red	Portal fibrosis Perivenular fibrosis Pericellular fibrosis Bridging fibrosis Nodular fibrosis Cirrhosis	Regions with fibrotic changes	Non-fibrotic liver regions

*AI*, artificial intelligence; *H&E*, hematoxylin and eosin; *N/A*, not applicable

### Evaluation of model performance

The validation was conducted through a pixel-level assessment, where AI models were evaluated on an independent test (validation) dataset comprising slides with pathologist’s manual annotations. This pixel-based evaluation aimed to assess the generalization capability of the models for future datasets, ensuring their robustness and accuracy in capturing histological features. Classification metrics, such as overall accuracy, macro-precision, and macro-sensitivity (macro-recall), were used to evaluate the performance of AI models. Accuracy was defined as the number of correct predictions divided by the total number of evaluations. Precision was calculated as TP/(TP + FP), and sensitivity was computed as TP/(TP + FN), where TP, FP, and FN are the number of true positives, false positives, and false negatives, respectively. In multiclass classification, each category forms its own positive class and combines other categories as the negative class, thus rendering several binary classifications. Macro-precision and macro-sensitivity are presented as arithmetic mean of individual binary precisions and of individual binary sensitivities, respectively. The performance metrics were calculated using the Aiforia platform.

The visualization and statistical analysis of quantification data output were performed using R (version 3.6.3). For data processing, the dplyr package was utilized, while data plotting was carried out using ggplot2. Confusion matrix statistical analysis was conducted using the caret package, and confusion matrix visualization was achieved with the cvsm package. Statistical analysis was performed using ggpubr. The *p* values were calculated using Student’s *t* test.

## Results

The biopsy slides of 116 patients (94 women and 22 men; mean age 59 years) were utilized. Two patients were biopsied twice at different time points before the treatment; therefore, 118 biopsies were used in the study. The biopsy material was split and embedded into two blocks for five biopsies; therefore, more than one slide was available for these biopsies. Overall, a total of 123 pairs of H&E- and Sirius red-stained AIH pre-treatment biopsy slides were included in the study.

Biopsy slides of patients were randomly assigned to training (*n* = 99) and test (*n* = 24) datasets. Baseline characteristics for the patients who provided biopsies were balanced between the training and test data sets (Table 2).

**Table 2:** Baseline characteristics of the patients who provided biopsies

Characteristic	Training set *N* = 93	Test set *N* = 23	Overall *N* = 116
Gender, *n*			
Male	18	4	22
Female	75	19	94
Age at diagnosis, years			
Mean	57	63	59
Median	61	67	62
Range, min–max	18–84	32–79	18–84

*max*, maximum; *min*, minimum; *N*, total number of patients in the arm; *n*, number of patients

### The pipeline for identification of AIH histology and model performance

To achieve the detection of AIH-related changes in liver biopsies, we developed a deep learning pipeline that consists of multiple deep learning models for different structures, features, and staining. A list of the final AI models with their layer structure and classes is presented in Table S1. Image analyses were run on the training and test set slides using the respective AI models. The models gave high test accuracies when evaluated on the separate test dataset (Tables 3 and 4).

**Table 3:** AI(H) performance on inflammation-focused tasks, H&E slides

Model	Overall accuracy (%)*		Macro-precision (%)^†^		Macro-sensitivity (macro recall) (%)^†^	
	Test	Training	Test	Training	Test	Training
Tissue detection	99.4	99.9	99.7	98.9	99.3	99.5
Microanatomy (portal area, lobular area, central vein)	88.0	97.5	94.2	98.3	93.7	96.6
Necro-inflammation (focal necrosis, interface hepatitis, confluent necrosis, pericentral necrosis, bridging necrosis, panacinar necrosis)	83.9	98.2	49.7	81.0	37.2	94.5
Portal inflammation	79.2	78.5	88.4	99.7	79.2	79.9
Immune cells (lymphocytes, plasma cells, macrophages, eosinophils, neutrophils)	72.4	83.6	86.9	91.8	85.2	91.8
Bile duct damage	81.7	90.3	91.3	95.4	90.3	95.0

^*^Overall accuracy is a standalone metric that measures how well machine-learning models perform in multiclass classifications. It denotes the ratio of correct predications; for example, for a three-category (category A, B, and C) classification task, overall accuracy is calculated as the sum of correct predications on category A, B, and C divided by the grand total

^†^Precision and sensitivity (also called recall) are paired metrics (which means that they cannot be used individually) that measure how well machine-learning models perform in classification tasks. In binary classification, precision is calculated as TP/(TP + FP), and sensitivity is computed as TP/(TP + FN). In multiclass classification, each category forms its own positive class (and combines other categories as the negative class) and thus renders several binary classifications. Macro-precision and macro-sensitivity are arithmetic means (average) of individual binary precisions and of individual binary sensitivities, respectively

*AI(H)*, artificial intelligence for hepatitis; *FP*, false positive; *FN*, false negative; *H&E*, hematoxylin and eosin; *TP*, true positive

**Table 4:** AI(H) performance on fibrosis-focused tasks, Sirius red slides

Model	Overall accuracy (%)*		Macro-precision (%)^†^		Macro-sensitivity (macro recall) (%)^†^	
	Test	Training	Test	Training	Test	Training
Tissue detection	99.4	99.9	99.8	99.3	99.0	99.8
Microanatomy (portal area, central vein)	94.0	97.0	67.0	92.4	65.9	83.7
Fibrosis (portal fibrosis, perivenular fibrosis, pericellular fibrosis, nodular fibrosis, cirrhosis)	87.6	97.2	73.3	96.2	68.3	95.1

^*^Overall accuracy is a standalone metric that measures how well machine-learning models perform in multiclass classifications. It denotes the ratio of correct predications; for example, for a three-category (category A, B, and C) classification task, overall accuracy is calculated as the sum of correct predications on category A, B, and C divided by the grand total

^†^Precision and sensitivity (also called recall) are paired metrics (which means that they cannot be used individually) that measure how well machine-learning models perform in classification tasks. In binary classification, precision is calculated as TP/(TP + FP), and sensitivity is computed as TP/(TP + FN). In multiclass classification, each category forms its own positive class (and combines other categories as the negative class) and thus renders several binary classifications. Macro-precision and macro-sensitivity are arithmetic means (average) of individual binary precisions and of individual binary sensitivities, respectively

*AI(H)*, artificial intelligence for hepatitis; *FP*, false positive; *FN*, false negative; *TP*, true positive

**Fig. 2:**
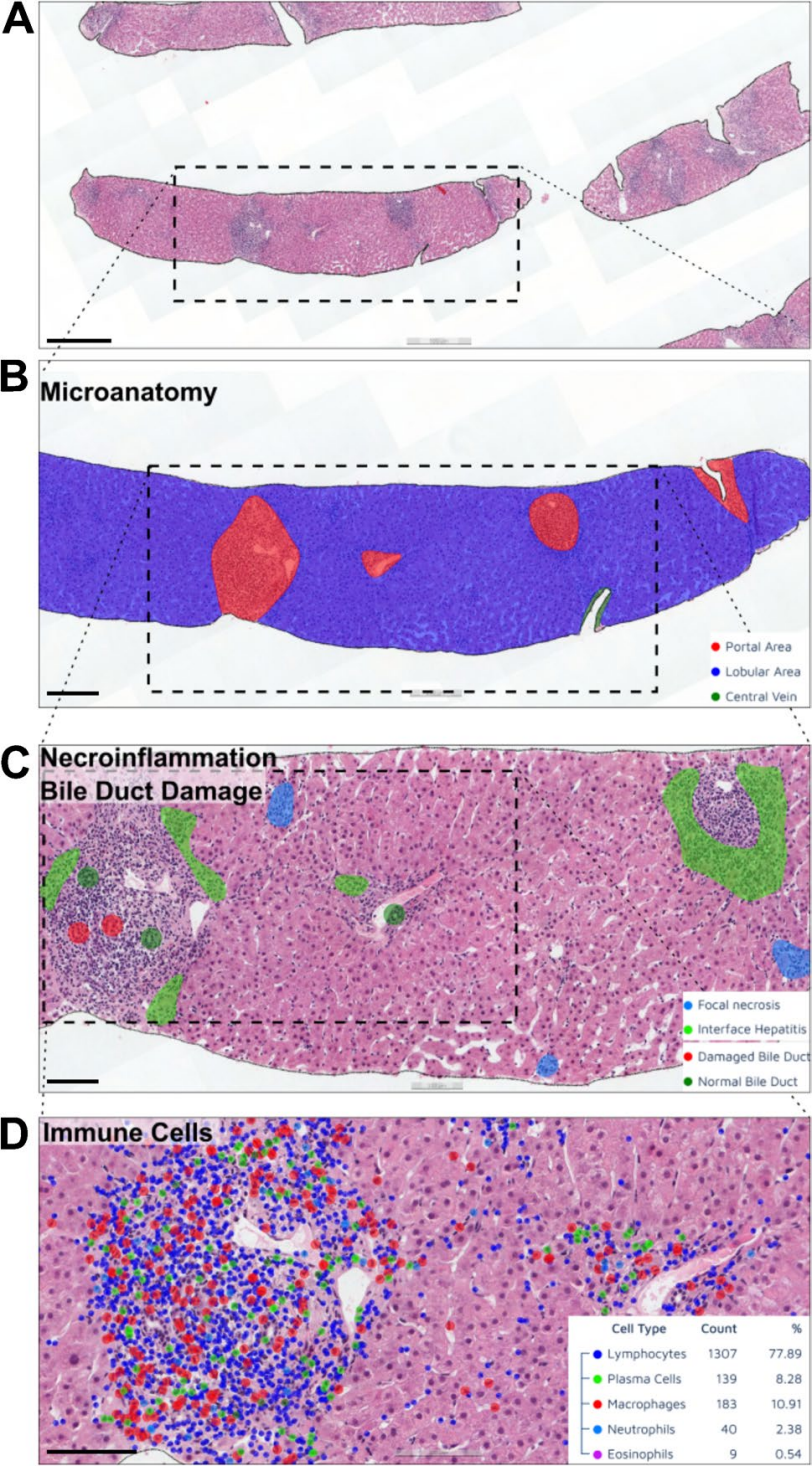
AI(H) predictions on H&E slides. The analysis of images is a multi-step process. **A** H&E-stained WSIs are used for detections (scale bar is 500 µm). **B** The AI model begins with segmenting liver tissue into its normal micro-structures, such as portal area, parenchyma, and central vein (scale bar is 500 µm). **C** The relevant necroinflammation features are detected in hepatitis landscape (scale bar is 250 µm). **D** Total five classes of immune cells (lymphocytes, plasma cells, macrophages, neutrophils, eosinophils) are detected and classified in all over the liver tissue. (scale bar is 200 µm). AI(H), artificial intelligence for hepatitis; H&E, hematoxylin and eosin; WSI, whole-slide image

We began with the segmentation of whole liver tissue area against background space. The tissue segmentation (foreground vs background) models showed excellent performance for both H&E- and Sirius red-stained slides, with over 99.4% accuracy. Following this, we set up semantic segmentation AI models for liver microanatomy which segments liver tissue into parenchyma, portal area, and central vein regions (Fig. 2). The pixel-level accuracy of the H&E model was 88.0% (Table 3), while that of Sirius red was 94.0% (Table 4).

We trained necroinflammation models on H&E slides to detect and classify elementary lesions of hepatitis such as interface hepatitis, focal necrosis, focal confluent necrosis, perivenular necrosis, bridging necrosis, and panacinar necrosis classes (Fig. 2). The overall accuracy of the necroinflammation segmentation model was 83.9% (Table 3). However, errors in the predictions mostly stemmed from lesions that were correctly classified but did not perfectly align with the ground truth annotations (Sup. Figure 1A-B). The portal inflammation model first detected the portal regions with inflammation, then consecutively graded them in a three-tier system: mild, moderate, and severe portal inflammation. The model had 79.2% accuracy (Table 3).

**Fig. 3:**
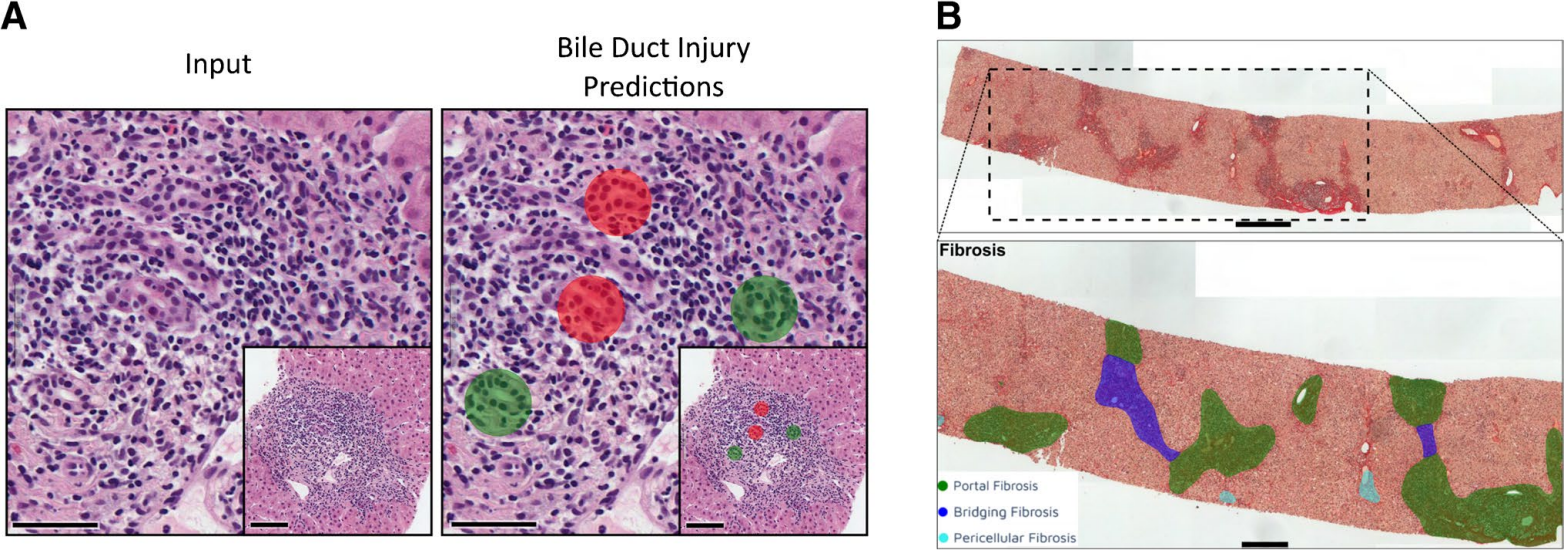
AI(H) predictions on Sirius red slides. **A** The bile duct damage can be detected even in highly inflamed of portal areas. (red injured bile duct, green normal bile duct) (the scale bars are 100 µm). **B** The Sirius red slides are analyzed for detecting the fibrosis-related changes. The fibrosis model was trained to segment portal fibrosis, perivenular fibrosis, pericellular fibrosis, bridging fibrosis, nodular fibrosis, and cirrhosis on slide images (top and bottom scale bars are 500 µm and 250 µm, respectively)

The immune cell classification model was designed to detect, classify, and quantify lymphocytes, plasma cells, macrophages, eosinophils, neutrophils, along with acidophil bodies (Fig. 2). To train and test the network, a total of 7868 annotations of immune cells were generated across all the datasets. The model accuracy rate for detection and classification of the immune cells was 72.4% (Table 3**,** Sup. Table 1). However, errors were observed primarily in densely inflamed regions, where the individual immune cell borders could not be easily differentiated (Sup Fig. 1C). Bile duct injury model detects and classifies bile ducts into “normal” and “damaged” categories. Bile duct injury was described as epithelial infiltration by mononuclear inflammatory cells, epithelial damage, and malformed, tortuous or irregularly shaped bile ducts (Fig. 3A) [8]. The accuracy of the model was 81.7% in the test dataset (Table 3). The result of the model showed that 69.5% (66/95) training set biopsies and 65.2% (15/23) test set biopsies (68.6% overall) had bile duct damage.

Fibrosis-related features were trained and tested on Sirius red-stained slides. The fibrosis model consists of portal fibrosis, perivenular fibrosis, pericellular fibrosis, bridging fibrosis, nodular fibrosis, and cirrhosis classes (Fig. 3B). Portal areas without fibrosis were left in the background. The overall accuracy of the model was 88.0%. While the model’s predictions often showed decent alignment with the ground truth annotations, errors in both the detection and classification of fibrotic lesions were observed in some regions (Sup Fig. 1D).

To get a deeper insight of AIH pathology, several AI models were combined to observe unique features together. For example, the combination of the necro-inflammation and bile duct models is shown in Fig. 2. The visual overlay output can assist pathologist for detection of particular lesions or evaluating the spatial relationship to detect the hotspot regions.

### Quantitative analysis of AI(H) predictions: comparison with pathologists’ evaluation

We conducted an additional analysis to further explore the utility of the AI-based image analysis tool. This analysis aimed to investigate the potential correlation between the AI(H) predictions and the pathologists’ assessments in terms of histological grading and staging of AIH biopsies. Quantification data obtained from the computational analysis were exported and compared among different grading feature groups as determined by the pathologists’ evaluations.

**Fig. 4:**
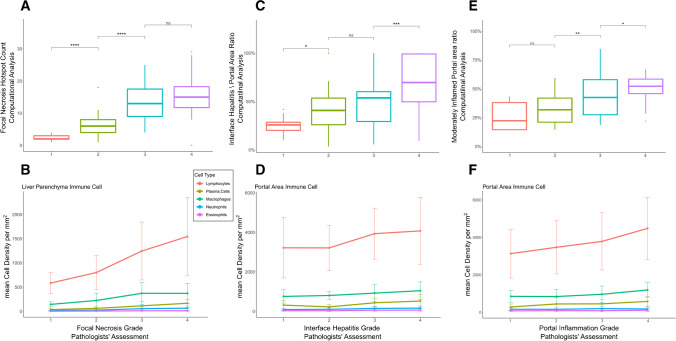
Comparison of histological features and AI(H) quantification results. **A** The maximum count of focal necrosis in 4 μm^2^ was compared between focal necrosis scores according to the Ishak scoring system. The AI(H) results are in line with the pathologists’ evaluations. **B** Moreover, the immune cell density in the portal area for the five cell types shows a noticeable increase in parallel with interface hepatitis scores. **C** Additionally, the immune cell density in the liver parenchyma for five cell types (lymphocyte, plasma cell, macrophage, eosinophil, and neutrophil) shows a clear increase in inflammation with focal necrosis counts. **D** The moderate-level portal inflammation ratio was compared between portal inflammation scores according to the Ishak scoring system. The AI(H) results indicate an increase in the ratio with higher scores. **E** The maximum ratio of the length of the portal area to the circumference of the portal area by interface hepatitis was compared across interface hepatitis scores. The AI(H) results exhibit an increase in the ratio with higher scores. **F** Furthermore, the immune cell density in the portal area for the five cell types displays a distinct increase with increasing portal inflammation scores

For focal necrosis, we compared the maximum count of focal necrosis in 4 µm^2^ between different focal necrosis scores (0–4 according to the Ishak scoring system). The results from AI(H) demonstrated a clear increase in focal necrosis counts with higher focal necrosis scores (Fig. 4A). Additionally, we observed a concurrent increase in immune cell density in the liver parenchyma for five different cell types along with focal necrosis counts (Fig. 4B).

In the case of interface hepatitis, we analyzed the maximum ratio of the length of the portal area to the circumference of the portal area for each biopsy, based on interface hepatitis scores. To calculate the circumference of the portal area affected by interface hepatitis, we utilized the portal area predictions from the microanatomy model as the denominator of the calculation. Our findings revealed a consistent increase in the ratio with higher interface hepatitis scores, as demonstrated by the AI(H) results (Fig. 4C). Similarly, the immune cell density in the portal area for the five cell types showed a noticeable increase corresponding to the severity of interface hepatitis (Fig. 4D).

Lastly, we investigated portal inflammation by comparing the moderate level portal inflammation ratio between different portal inflammation scores (0–4 according to the Ishak scoring system). The AI(H) predictions exhibited an incremental pattern with increasing portal inflammation scores (Fig. 4E). Furthermore, the immune cell density in the portal area for the five cell types exhibited a parallel increase in relation to portal inflammation scores (Fig. 4F).

The results of this comparative analysis provide insights into the concordance between AI-based predictions and expert pathologists’ evaluations, thereby highlighting the potential clinical value of AI(H) in enhancing the accuracy and consistency of AIH assessment.

### Utilization of AI(H) outputs for AIH histopathology assessment

We explored the potential of model’s quantification outputs for stratifying liver biopsies according to the latest consensus recommendations for histological criteria of AIH from the International AIH Pathology Group [6]. Among the samples analyzed, the 29/119 (24.4%) exhibited a “Portal hepatitis pattern” (Sup. Figure 2A). All the portal hepatitis samples showed either lobular hepatitis or interface hepatitis, thus 29/29 (100%) were classified in the likely category. On the other hand, 90/119 (76.6%) of biopsies were classified as “Lobular hepatitis pattern” (Sup. Figure 2A). Among these, 74/90 (82.2%) samples demonstrated the presence of at least one of the following: interface hepatitis, portal fibrosis, or lymphoplasmacytic inflammation, classifying them as likely for AIH, while 19/90 (17.8%) biopsies within the lobular hepatitis category were considered possible for AIH.

Subsequently, we compared the predictions of AI(H) against the pathologist’s diagnosis based on the latest consensus recommendations for histological criteria of AIH. The predictions of AI(H) showed 88.2% accuracy in classifying AIH biopsies into “likely” and “possible” categories (Supplementary Fig. 2B). Misclassification within the “likely” category was primarily due to overdiagnosis of interface hepatitis, indicating potential for improvement in feature differentiation (Supplementary Fig. 2C). Conversely, misclassification in these samples was also influenced by factors like discoloration from long archive time and severe parenchymal necrosis, underscoring the importance of sample quality for accurate AI-based diagnosis (Supplementary Fig. 2D). Collectively, these results demonstrate potential use cases of the quantification output of AI(H) in pathologist’s evaluations.

While the AI(H) model has not been trained to identify features suggestive of other liver diseases, nor has it been systematically validated for clinical diagnosis performance, it is suboptimal for this purpose. Nonetheless, our experiment illustrates its versatility in providing quantification data for various applications.

### Detection of chronic hepatitis features in non-AIH liver biopsies by AI(H)

In light of the similarities in elementary lesions observed in hepatitis biopsies, including focal necrosis and interface hepatitis, we explored the detection performance of AI(H) for chronic hepatitis features in samples diagnosed with other acute and chronic hepatitis conditions. Specifically, we examined liver biopsies diagnosed with drug-induced liver disease with acute lobular hepatitis and HCV- and HBV-chronic hepatitis.

While AI(H) was not initially developed for analyzing non-AIH liver biopsies, our observations reveal its capability to recognize hepatitis elementary lesions, such as focal necrosis, interface hepatitis, and portal fibrosis, alongside immune cells and bile duct damage (Supplementary Fig. 3). Although the model demonstrated decent performance across various tasks, we noted incidental errors, such as false bile duct damage classification in inflamed regions and underestimation of immune cells. Of particular note, while many interface hepatitis regions were detected correctly, some detections were smaller than the lesions themselves. Overall, these findings suggest that with fine-tuning using annotations for each disease, the model holds potential to be utilized in other liver diseases.

## Discussions

The semi-quantitative manner of the AIH pathological assessment brings an inherent degree of inter- and intra-observer variability in AIH assessment. AI(H), a digital AI tool developed primarily for the granular quantification of biopsy images, serves to enhance the analysis process by providing precise, consistent, and rapid analysis of AIH histology While it can help ease the challenges faced by pathologists, it is essential to emphasize that AI(H) is intended as an adjunctive tool rather than a standalone diagnostic solution. In addition, it can provide precious quantification data from H&E slide analysis. Its primary function is to augment pathological analysis and offer valuable quantification data from H&E slide analysis, ultimately supporting but not replacing the expertise and clinical judgment of trained professionals.

While current advances in computational pathology of neoplastic diseases are producing positive findings, more challenging non-tumor pathologic diseases have, for the most part, been omitted owing to the complexity of diagnosis (i.e., clinical, laboratory, and histological features). There are an increasing number of studies in hepatology that use pathology slide images for AI-based model development [21]. The majority of the non-neoplastic studies are focused on non-alcoholic steatohepatitis [21], and only a few computational pathology studies are focused on other liver diseases, such as viral hepatitis [22], liver transplantation [23], and primary sclerosing cholangitis [24]. To our knowledge, this is the first AI tool developed for the analysis of AIH histology. The AIH immune ecosystem consists of a varying number of mostly chronic inflammatory cells whereby the frequency and alteration of certain immune cell types are related to disease activity and response to treatment. The computing capacity of AI provides a larger amount of information from biopsies that is not routinely possible by pathologist evaluation, such as identifying, categorizing, localizing, and counting large quantities of immune cells. Thus, AI opens new avenues for evaluation and quantification of liver inflammation.

An unmet need exists in hepatopathology for fast, reproducible, and quantitative diagnosis [25]. The AI(H) algorithm in our study accurately predicted key AIH components such as portal lymphoplasmacytic infiltrate, interface hepatitis, lobular activity, and fibrosis with high efficiency, in classifying and counting cells and tissues. To address the complex nature of AIH histology, a cascade of AI models targeted distinct challenging histological features. The AI(H) algorithm accurately identified key AIH features with high efficiency but faced challenges in immune cell detection. There can be several reasons for this: for immune cells, the training data are more noisy and not representative enough, the true effective sample size (not the number of training biopsy slides) is smaller, and the combined detection of classification task of single immune cells is more complicated. Hence, the accuracy for immune cells is lower than the accuracy for microanatomy. These models, targeting elementary lesions of chronic hepatitis, offer adaptability to other scoring systems (such as METAVIR [26]), potential future changes in AIH assessment [6], or the evaluation of other chronic hepatitis biopsies, [7] like viral hepatitis. Bile duct damage in AIH is acknowledged to be confusing, and most of the time it is an overlooked histological feature. Therefore, ambiguity of their significance remains unresolved. The presence of bile duct injury as an incidental histological feature in AIH was found in various proportions in the reported literature, such as 24%, [27], 72%,[28], and 83% [8]. AI(H) defined the proportion of biopsies with bile duct injury as 68.6% in our dataset, which is closer to Kuiper et al.’s result (72%) [28]. This discrepancy may stem from the changing AIH diagnosis criteria over time. With the leverage of AI-based image analysis approaches, the under-investigated fields of pathology could be revealed, and histological feature relationships could be elaborated.

Interpretability and visualization are important aspects in the computational pathology field. In clinical practice, pathologists can significantly benefit from the overlay of AI model’s predictions for their qualitative self-evaluation of the biopsies along with quantitative output. AI(H) makes pathologist-level evaluation of hepatitis and may be able to guide pathologists to disease specific features on biopsy images. However, this study faces limitations: it used biopsies solely from one institution, with annotations by a single pathologist; AIH liver biopsies with highly disrupted architecture or slides enriched with necro-inflammation reduced the accuracy of the AI model predictions. In addition, although the grading and staging of the liver biopsies are based evaluation of two pathologist, because the evaluations were set in evaluation and consensus of two pathologists, the effect of inter-observer variability could not be assessed in the study. While the AI(H) model has high accuracy, there were a few mislabeled features specific to areas where fibrosis staging was high. Additionally, only Sirius red-stained slides were used for the assessment of connective tissue stains for collagen and training the model for the fibrosis. Although the most recent consensus states that the method of choice depends on the experience and routine protocols established in each center, evaluation of elastic fiber staining (such as such as orcein, Victoria Blue or Elastic van Gieson) is important to distinguish recent collapse from longstanding fibrosis [6]. It would be beneficial of incorporation of an additional elastic fibrin staining in liver fibrosis assessment in future studies.

Since the diagnosis of AIH requires combination of microscopical finding along with clinical story and laboratory values, being a solely image-based analysis tool is a significant limitation for AI(H) for its diagnostic tool. Additionally, differential diagnosis of microscopical findings from other liver diseases is important aspect in AIH diagnosis. Exclusion of coexisting liver diseases limits the model’s real-world diagnostic applicability [29]. Moreover, the AI(H) model’s reliance solely on slide images, without clinical or lab data, restricts its capacity for comprehensive differential diagnosis. Future improvements necessitate a larger, diverse training set to enhance diagnostic accuracy and minimize misdiagnoses of other liver conditions as AIH. Calculating sensitivity and specificity using AI(H) is imperative for reliable clinical predictions. Additionally, multimodal AI models emerge for other fields of computational pathology for making mode precise predictions of patient prognosis by an integration of multiple patient information, such as pathology, radiology and genomic [30–32]. Similar study designs which combine pathology images, laboratory results, and patient history, could yield a diagnostic tool for AIH diagnosis.

AI(H) helps in classifying the different regions of AIH histology by providing a granular, quantifiable, and consistent analysis of AIH pathology. It was accurate and efficient in predicting various morphological components of AIH biopsies and has potential to aid in AIH microscopical assessment. The AI-based image analysis tool’s capacity to deliver reproducible and centralized results for biopsy evaluation makes it particularly valuable for clinical trials. This potential advantage constitutes a significant strength of the present study. However, AI(H) needs further studies to address this study’s limitations. In addition, the classical advantages of computational methods (e.g., high reproducibility, speed, and quantitative capabilities) make AI(H) a promising interpretable computational pathology tool to facilitate histological recognition in the era of augmented pathology.

## Supplementary Information

Below is the link to the electronic supplementary material.

Supplementary file1 (DOCX 6109 KB)

## Data Availability

The data that support the findings of this study are available from the corresponding author upon reasonable request.

## Abbreviations

AIH: Autoimmune hepatitis
AI: Artificial intelligence
AI(H): Artificial intelligence for autoimmune hepatitis
CNN: Convolutional neural networks
FN: False negative
FP: False positive
H&E: Hematoxylin and eosin
TP: The number of true positives
WSI: Whole-slide image
