# Supplementary material for: A deep-learning-based model for assessment of autoimmune hepatitis from histology: AI(H)
Source: Virchows Arch. 2024 Jun 15;485(6):1095–105. doi: 10.1007/s00428-024-03841-5 (PMC11666607; doi:10.1007/s00428-024-03841-5)

Supplementary Table 2. Cell type specific performance of immune cell detection and classification.

| Immune cell type | Precision | Sensitivity | F1 Score |
| --- | --- | --- | --- |
| Lymphocytes | 89.97  (2296 / 2552) | 87.80%  (2296/2615) | 88.87% |
| Plasma cells | 78.14%  (454/581) | 79.93%  (454/568) | 79.03% |
| Macrophages | 80.23%  (211/263) | 75.09%  (211/281) | 77.57% |
| Neutrophils | 73.63%  (67/91) | 83.75%  (67/80) | 78.36% |
| Eosinophils | 90.24%  (74/82) | 79.26%  (75/97) | 82.68% |

**Supplementary Figures**

**Supplementary Figure 1: Examples of Errors in Model’s Predictions**

**(A)** The most common observed error for interface hepatitis detection was the failure to make perfectly aligning predictions. The predictions (right pane, solid green) exhibit interruptions, while the ground truth is continuous (left pane, green dashed line). **(B)** The most common error for focal necrosis was the conglomeration of multiple closely located focal necrosis foci. **(C)** The most common errors in the immune cell model stemmed from the detection of cells in densely inflamed regions where the borders of cell nuclei cannot be identified clearly. **(D)** While the fibrosis model demonstrated overall decent performance for the detection of common fibrotic features, some important features (such as bridging necrosis in the example image, arrowhead) were missing in some images.


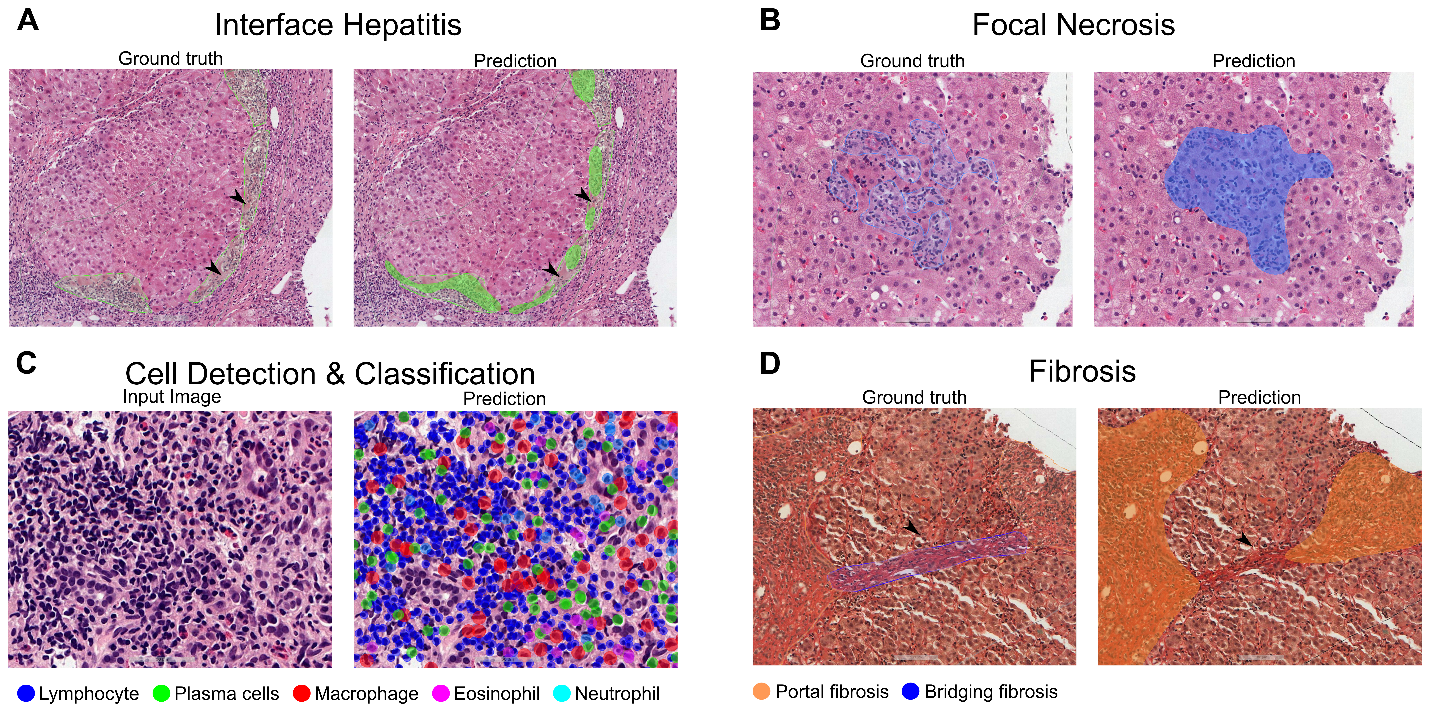


**Supplementary Figure 2: AIH diagnostic category classifications**

**(A)** Biopsies were classified into AIH diagnostic categories based on Consensus recommendations for histological criteria of autoimmune hepatitis from the International AIH Pathology Group. **(B)** The accuracy of the classification predictions against pathologists' diagnosis was 88.2%. (C) Misclassification of samples in the likely category was observed, primarily due to overdiagnosis of interface hepatitis. (D) Conversely, misclassification of likely category samples was observed in conditions such as discoloration due to long archive time, fragmented samples, and severe parenchymal necrosis, which rendered the model unable to accurately analyze the tissue.
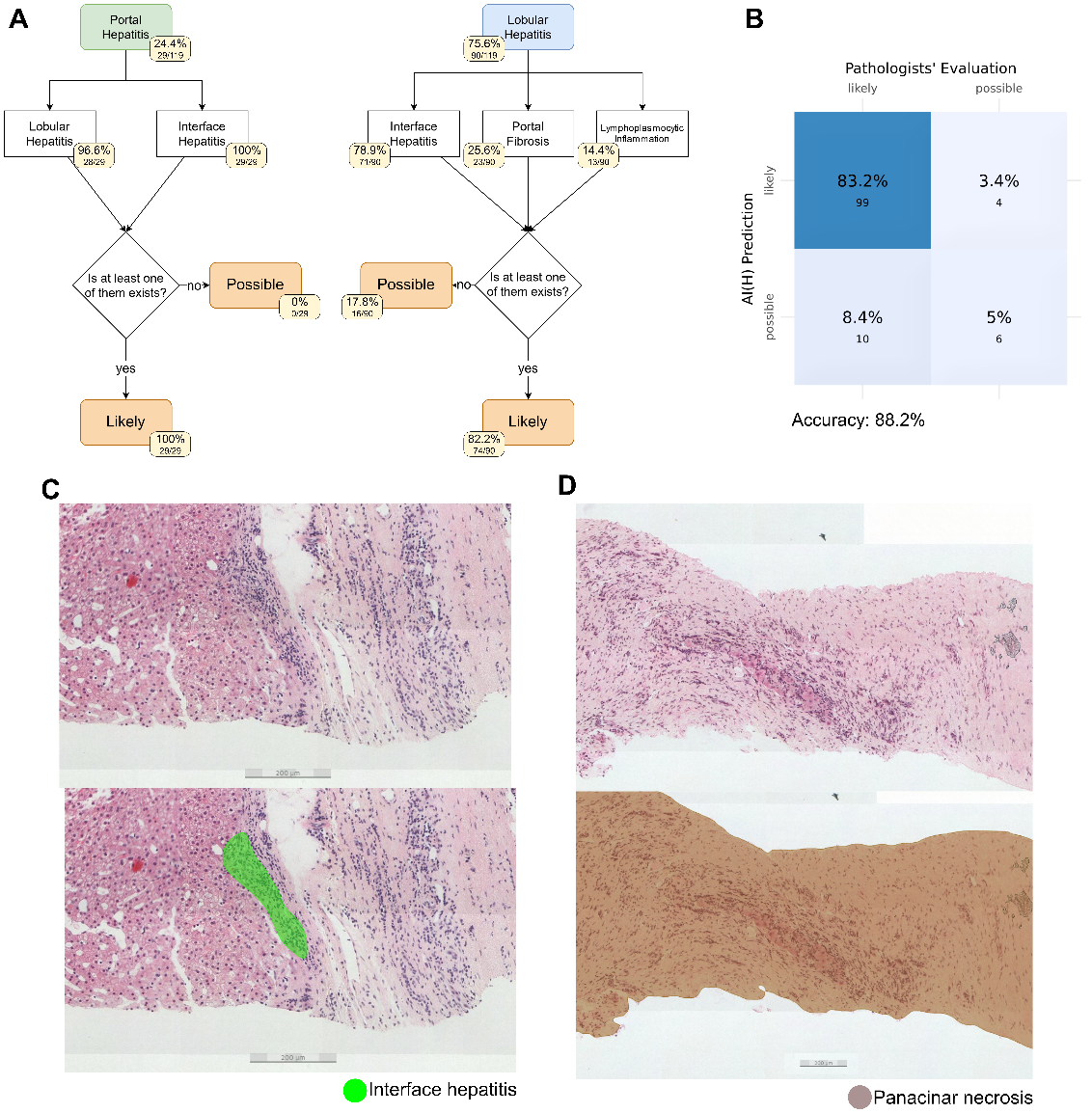


**Supplementary Figure 3: Exploration of Performance on Other Hepatitis biopsies**

Although AI(H) was not trained on liver biopsies without autoimmune hepatitis, it demonstrated overall promising performance in detecting various hepatitis-related lesions and cell detections.


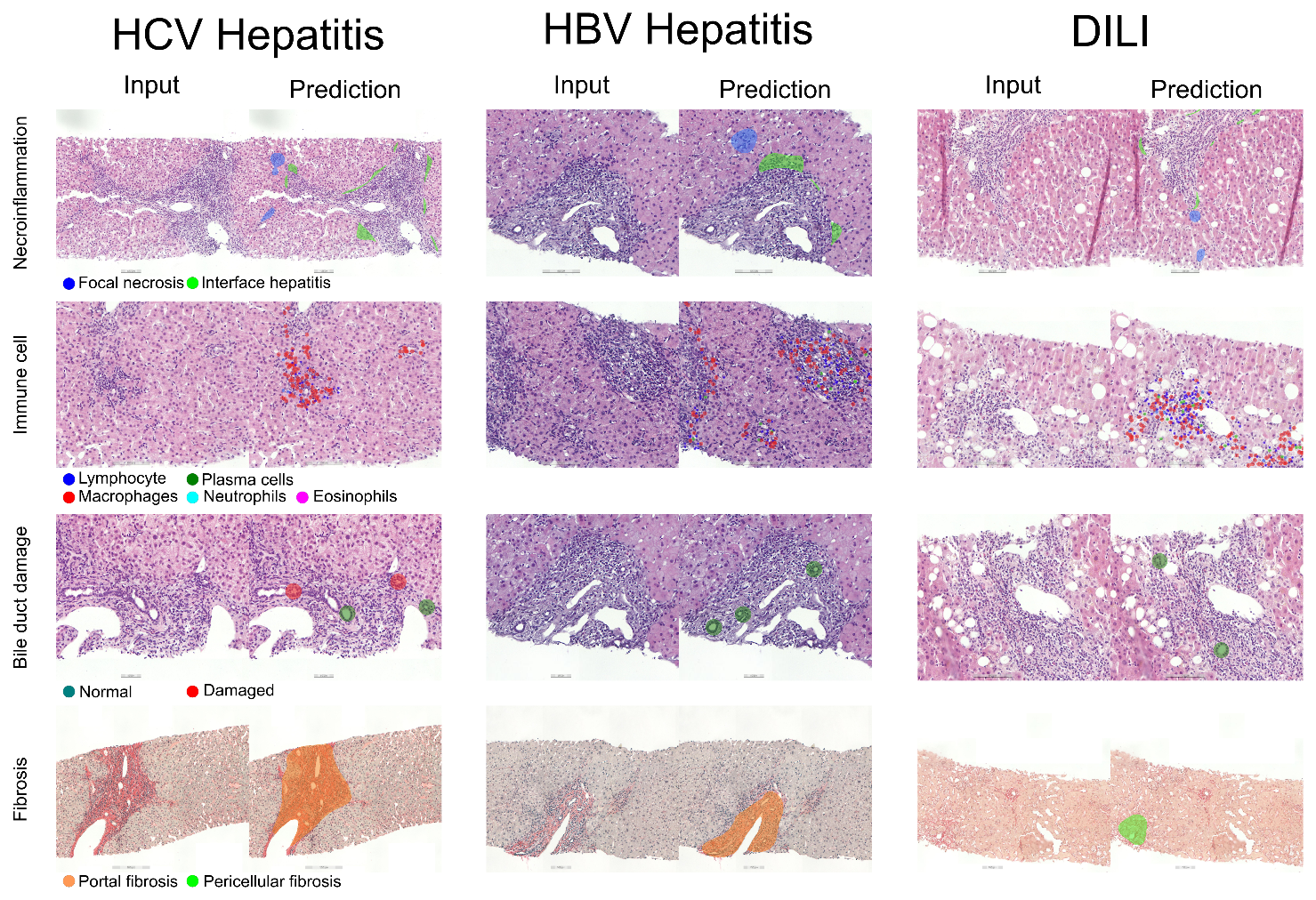

Supplement: Supplementary file 1 — Supplementary file1 (DOCX 6109 KB) [file 428_2024_3841_MOESM1_ESM.docx]
